# Whose city? Which sociality?

**DOI:** 10.1080/02723638.2021.2007665

**Published:** 2022-01-17

**Authors:** Jennie Middleton, Farhan Samanani

**Affiliations:** a School of Geography and the Environment, University of Oxford, Oxford, UK; b Max Planck Institute for the Study of Religious and Ethnic Diversity, Göttingen, Germany

**Keywords:** Urban, social infrastructure, sociality, austerity

The concept of “social infrastructure” has enjoyed a quick ascendancy, coined by sociologist Eric Klinenberg in a 2013 *New Yorker* article, elaborated in his 2018 book, and popularized within human geography by a 2019 article by Alan Latham and Jack Layton – the editors of this special issue. Klinenberg, arguably best known for his study of the 1995 Chicago heatwave (2002), offers a striking illustration of the value of social infrastructure. During the heatwave – an event that claimed 739 lives – the pattern of deaths largely followed the city’s geography of poverty and racial segregation. But there were exceptions. Klinenberg (2013, n.p.) compares “Englewood and Auburn Gresham, two adjacent neighborhoods […] both ninety-nine per cent African-American, with similar proportions of elderly residents. Both had high rates of poverty, unemployment, and violent crime.” Yet, Englewood experienced one of the city’s highest death rates, Auburn Gresham, one of the lowest. The difference, argues Klinenberg, was Auburn Gresham comparatively robust social infrastructure – “the people, places, and institutions that foster cohesion and support” (Klinenberg, 2013). He quotes Betty Swanson, a long-time resident, who recalls: “During the heat wave, we were doing wellness checks, asking neighbors to knock on each other’s doors […] The presidents of our block clubs usually know who’s alone, who’s aging, who’s sick. It’s what we always do when it’s very hot or very cold here” (Klinenberg, 2013). The concept of social infrastructure usefully captures both the ways in which social ties are facilitated by infrastructural forms, and the ways in which such ties extend and reshape the “capacities” of citizens, together (Latham and Layton 2021). As the contributors to this special issue highlight, with clarity and urgency, the absence or presence of such socially mediated capacities can play a pivotal role in justice and well-being. As Klinenberg reminds us, they can even literally make the difference between life and death.

The contributions to this special issue illustrate both the flexibility and the analytic utility of the concept of social infrastructure – which is drawn on to make sense of everything from the ways in which domestic lighting practices in East London can be used to cultivate intimate and hopeful forms of sociality (Ebbensgard 2020), to how an inner-city service hub in Osaka creates forms of “dense social connection” (DeVerteuil et al 2020: 4), which simultaneously extend and circumscribe the capacity of voluntary organizations to care for those in need and transform the city, respectively. As the concept of social infrastructure gains traction, we hope to offer a series of reflections that build on the contributions here by considering the future use of the term whilst highlighting potential areas of further investigation. Drawing upon the different papers, we raise six related considerations. First, we highlight the value of talking about sociality not as a generic domain, but in terms of contrasting, even, sometimes contending, “modalities” of sociality. Second, this can prompt us to trace the different – partly overlapping, partly disjunctive – geographies of different modes of sociality, and of the infrastructures that sustain them. Third, following on this, even when we come across forms of sociality that are positively valued or championed by some, we should resist treating these forms of sociality as universally valued. Fourth, we might ask how social infrastructures produce additional effects, across a range of scales, beyond the cultivation of sociality. Fifth, as with any conceptual coinage, there is a risk that the concept of social infrastructure risks dividing urban life into a number of discrete, even opposed domains – a risk that, in this case, would undermine some of the very potential of the concept to point to the importance of social entanglements. Sixth, and finally, we ask whether there is a need to imagine and investigate forms of social infrastructure that do not simply promote sociality, but which, given the plurality of social life, mediate between contending forms of sociality and clashing claims to space.

In earlier formulations, “social infrastructure” has often imagined “the social” as a relatively generic and circumscribed domain – internally similar and externally bounded. This understanding, for instance, is reflected in Klienberg’s formulation quoted above where social infrastructure entails “the people, places, and institutions that foster cohesion and support”. Here, “sociality” is characterized as ties that bind people to others, and create an investment in their welfare. It is also taken as something that has a distinct, uneven distribution – something that can be more or less present within certain places, or across certain relationships. Both these characterizations can be productively examined further, in ways that would enable us to enrich and extend our understanding of social infrastructure and its value.

These two concerns – pertaining to “what” we imagine sociality to entail and do, and “where” we imagine it to occur – are deeply intertwined. Nonetheless, we can better clarify each one by approaching them separately. Regarding the first, building upon Kleinberg’s framing where sociality is oriented around cohesion and support, we might identify different modalities of coming together or supporting others. In their own empirical contribution to this special issue, exploring the social infrastructure and public life of Finsbury Park, North London, Latham and Layton (2021, p. 3) argue that it “is essential to think carefully about the different registers that make up the social in social infrastructure”. In surveying the different uses of Finsbury Park – which, in different cases, unfold separately to one another, reinforce one another, or clash – they name six distinct “registers” of sociality: “*co-presence; sociability and friendship; care and kinship; kinaesthetic practices; carnivalesque and collective experience; and civic engagement*” (2021, p. 11, Emphasis in original). Yet as soon as we pluralize the social, and begin considering different modalities rather than treating it as a generic domain, then we must also consider the ways in which different forms of sociality work in harmony or in dissonance with one another, and how they operate to support or constrain, enrich or diminish life, for different actors. “Cohesion and support” for whom, and of what sort?

Meanwhile, the concern of “where” we locate sociality, has to do in part with clarifying our epistemological apparatus and ontological commitments – our ways of knowing, and our prior assumptions about the world. As the editors of this special issue highlight in the introduction, for some, including human geographers, the social does not exist as a discrete domain – rather it serves as an encompassing term for almost any system of relations. There are different ways of approaching this claim. For instance, in the allied discipline of anthropology, many anthropologists would recognize this as a self-conscious epistemological commitment – a way of seeing that allows for insight into human life, but which can also be reflexively subject to scrutiny and reworking in big ways and small (Strathern, 2020). Actor Network Theorists may take the argument a step further, arguing for a thoroughly social ontology, where “sociality” is the basic stuff of reality (Latour, 2005). The use of “social infrastructure” within human geography is still fairly recent, and so questions of how the concept dovetails with particular epistemological and ontological commitments provide a productive avenue for future research. We see the potential in further specifying how “sociality”, as imagined by theorists of social infrastructure, overlaps with or breaks from other dynamics, within physical and imaginative space. In this issue, just as Prytherch contrasts circulation and sociality, Latham and Layton (2021, p. 3) argue against only seeing public space “through the frame of the political”. If “circulation”, “politics”, and “sociality” are being framed as different domains, how do these domains map onto and break from one another? Moreover, if we are to consider them as such, what sorts of epistemological and ontological commitments might underwrite such multi-dimensional knowledge?

These questions point toward a third consideration, relating to how “sociality” is valued. By pluralizing sociality, and looking at different modalities, and by tracing it geographically in relation to other domains such as politics, we also end up with a more ambivalent notion of the social. Forms of sociality that may be enriching for some may prove exclusionary or detrimental toward others – a point made in different ways by several contributors to this special issue (Campbell et al; DeVerteuila et al; Latyon and Latham; and Prytherch) Returning again to Klinenberg’s focus on “cohesion and support”, we can identify a strong kinship with the notion of “social capital” (perhaps not by coincidence, given Klinenberg’s own background as a sociologist). Within sociology itself, this concept has generated an animated debate and prompted important interventions that have highlighted that social capital does not always (only) enhance interpersonal relationships and networks but can also have negative effects, including contributing towards the oppression and marginalization of certain groups (see Portes, 1998 for a seminal critique; and Villanlonga-Olives and Kawachi, (2017) for a recent overview). Similarly, Iris Marion Young (1986) has influentially highlighted how notions of community, which demand that members cultivate close forms of interpersonal understanding and interdependency, can work to suppress and diminish forms of difference and freedom. These debates resonate with the explorations of social infrastructure here. At stake in these critiques is not simply a clash between different modes of sociality, then, but a more fundamental ambivalence in what “cohesion and support” actually does for different citizens. For example, recent work emerging from critical walking studies (see Middleton, 2022; Springgay & Truman, 2019) has shown the power relations that can unfold from how people appropriate urban space on foot and the ways in which pedestrian social encounters are frequently either romanticized and underpinned by a series of positive assumptions or simply considered a benignly neutral aspect of contemporary urban life. Focusing on the mircro-politics of pedestrian encounters demonstrates how walking is not always a positive urban practice but can also be something to endure (see also Davidson, 2020). This ambivalence plays a meaningful role throughout the contributions here, yet we also see instances where contributors celebrate “social capital” (Layton and Latham), “bringing communities together” (Campbell et al) or “dense social connection” (DeVerteuila et al), in terms that presents sociality as innately positive. The challenge, as we see it, is for future research to keep a sharp focus on both the enriching and diminishing possibilities of different forms of sociality.

Fourth, and once again following on from this, is the question of how social infrastructures are implicated in wider patterns of power. Here, we note that a number of contributions to this issue highlight the ways in which social infrastructures not only generate (positively valued) modes of sociality but also different scalar relations that can work to uphold wider configurations of power. Thus, for instance, Ebbensgard (2020) explores how residential lighting practices in East London work to sustain forms of domestic intimacy and hope that make the city and the present liveable. At the same time as noting these valued effects, however, Ebbensgard also asks what role such practices might play in working to resist or extend urban inequality – whether they generate resistance or acquiesce, hope for a different future or simply desire to achieve a superior position within a stratified present. DeVerteuil et al’s (2020) study of a “service hub” in Osaka is even more pointed, highlighting how urban policy in Osaka has worked to carve out a great deal of autonomy for this hub, enabling it to create empowering forms of social infrastructure at a local scale while simultaneously constraining its transformative potential against the realities of urban poverty, or within urban policy, more broadly. These examples highlight the capacities of social infrastructures to generate multiple outcomes – beyond forms of sociality – and point to another productive avenue for future research, in investigating how these outcomes interact and counterbalance.

Fifth, and again relatedly, we would suggest that a certain caution is warranted in how and when we rely on the concept of social infrastructure to frame the subject under study, to avoid unduly fragmenting the urban. Of course, the naming of sociality as an important part of what infrastructures do, or ought to do, is valuable both for directing our normative and analytic attentions. There is a promise here of a more integrated and inclusive vision of the urban. Yet, perhaps counterintuitively, we would suggest that this same act of naming risks being part of an analytic and normative fragmentation of the urban, as much as it may be a part of widening our gaze. This threat of fragmentation is a product of how social infrastructures have thus far been approached – as a named domain, and as part of an exercise in dividing infrastructure, or the urban, into distinct domains (sociality, mobility, knowledge, health, and so on), which then only in the second instance can be thought of as interacting or overlapping. Here, the danger becomes that we direct our attention and imagination toward forms of social infrastructure that are defined in distinction or opposition to other forms of infrastructure, rather than searching for forms that blend different possibilities together.

An illustrative contrast comes from approaches informed by feminist thinking on care and positionality, which prioritize the processes of situated reasoning – grounded in interaction, skilled engagement, situated attentiveness, and ongoing moral accountability (Tronto, 1993) – above and beyond the drawing of abstract categories of judgment which are held to be the same, wherever they are applied. As we have argued elsewhere (Middleton & Samanani, 2021), such approaches remind us of two vital things: first that everyday life is full of attempts to negotiate between contending values, possible futures, and political conflicts; and secondly that analytical moves to abstract from a narrative, a vignette, a set of laws, a text, and so on, to say what it is really “about”, necessarily obscure and devalue these complex negotiations – foreclosing or overlooking countless movements of everyday potential that sustain the world and/or make it otherwise. When it comes to social infrastructures, by reifying this category, we may risk only attuning ourselves to, and championing that which has the scale, visibility, and concreteness associated with typically grand, systemic, “public” visions of infrastructure; we risk paying more attention to highways and community centers than we do to the ways in which black American women, for example, have shared ways of making “homeplaces” for generations that provide collective means of refuge, endurance, and dignity, against systematic forms of deprivation and denigration (Hooks, 1990).

Of course, as the aforementioned characteristics of careful knowing (interaction, skilled engagement, etc.) may suggest, forms of situated knowledge are often hard won, and it may well be unrealistic to expect that all judgments of urban or infrastructural policy and design are guided by such forms of knowing. Here, more categorical forms of knowledge can and sometimes must serve as useful heuristics. Yet we should always be mindful of what such categories are working to achieve: are they further carving up a map of the city into discrete, abstract domains – which are then taken in opposition to one another – or are they helping us attune ourselves to the dimensions of an unequal but inextricably entangled whole? Or, to put the point differently, even when thinking heuristically, it may help to stop and ask ourselves when it is useful to talk about “social” infrastructures, and when, instead, it becomes more helpful to shift frames and talk about infrastructure in other terms such as infrastructures of endurance, improvisation, accountability, and so on – in ways that clearly encompass sociality, but focus more on how it forms a part of broader assemblages and leads to particular political ends, rather than taking sociality as an end in and of itself. Indeed, DeVerteuil et al’s (2020) study of a “service hub” in Kamagasaki, Osaka, performs this sort of reframing – taking an interest in social infrastructure primarily as a matter of connectivity between services, and an open-access, non-judgmental ethos, and discussing this in more specific terms as *bypassed infrastructure*, which by dint of its overlooked and relatively structurally unincorporated position, is able to create distinctive new assemblages.

Finally, taken together, these strands point to questions of how to negotiate between different forms of sociality. A number of contributors engage with this issue from different angles. Campbell et al. raise questions about the geographical distribution of stewardship groups that maintain, mediate, and facilitate access to green spaces, and about how such groups can “address diversity, equity, and inclusion through their programs and organizational structures” (2021: 17). DeVerteuil et al. ask how forms of social infrastructure might specifically serve to cultivate counterpublics that foster both acceptance and the capacity for resistance (while expressing ambivalence over the potential of the service hub they study to do so). Prytherch is concerned with diverse, but often contending uses of street space, whilst Ebbensgaard grapples with the capacity of intimate socialites to both sustain hope and possibility and to foster acceptance of inequalities. Part of what is stake in these questions is how different forms of sociality, which offer different perspectives on the city, and which appropriate it in particular ways, might be situated in relation to one another. How does intimacy square with critical consciousness, and an awareness of urban entanglements, how might commitments to park stewardship also become commitments to justice and inclusion, what capacities ought to be cultivated for marginal citizens in order to navigate their marginality?

In their original elaboration of the concept of social infrastructure, Latham and Layton (2019, p. 8) argue that the “diversity of social infrastructure matters. People seek out a range of activities and communities and therefore require a range of facilities and spaces”. They likewise follow Ash Amin (2008) in arguing for the potential of social infrastructures to cultivate a sense of ‘urban surplus’ – encouraging trust, civility, encounter, and common purpose. A question for further research is the extent to which cultivating a diversity of infrastructural forms, and evoking a sense of social surplus is enough. Contributions in this issue point to the ways in which different socialites might sometimes involve processes of urban appropriation and transformation that diminish or cut against one another. At a time where austerity policies have guided national and urban policies across many democratic nations for the past decade, and where the broader global entrenchment of neoliberal economics has long fed into the residualization of public resources, it is entirely possible that these tensions may ultimately prove to be a problem of scarcity. This is to say, it is possible that with sufficient investment in social infrastructure, including the multiplication of different forms that might support a diversity of distinctly valuable socialites, these contradictory claims may well diminish or vanish all together. Yet we ultimately live on a finite and politically constrained planet – one where the prolific multiplication of social-infrastructural forms, at a scale commensurate with sustaining just, inclusive cities, may not be feasible. There is also a tricky normative question at stake here – whether the good city is more defined in terms of wide-reaching entanglements and interdependencies, that ensure forms of sociality take shape with regard to one another, or whether the good city is one which lets a thousand flowers bloom. Here too there is fertile ground, both for further inquiry and an ongoing conversation. This special issue highlights the rich potential for empirical investigations of social infrastructure to nurture such inquiry, and to open up future horizons.
